# Honokiol Affects Stem Cell Viability by Suppressing Oncogenic YAP1 Function to Inhibit Colon Tumorigenesis

**DOI:** 10.3390/cells10071607

**Published:** 2021-06-26

**Authors:** Dharmalingam Subramaniam, Sivapriya Ponnurangam, Satish Ramalingam, Deep Kwatra, Prasad Dandawate, Scott J. Weir, Shahid Umar, Roy A. Jensen, Shrikant Anant

**Affiliations:** 1 Department of Cancer Biology, University of Kansas Medical Center, Kansas City, KS 66160, USA; ksdlingam@gmail.com (D.S.); ponnurangamsivapriya@gmail.com (S.P.); rsatish76@gmail.com (S.R.); deep.kwatra0@gmail.com (D.K.); pdandawate@kumc.edu (P.D.); sweir@kumc.edu (S.J.W.); 2 Department of General Surgery, University of Kansas Medical Center, Kansas City, KS 66160, USA; sumar@kumc.edu; 3 Department of Pathology and Laboratory Medicine, University of Kansas Medical Center, Kansas City, KS 66160, USA; rjensen@kumc.edu

**Keywords:** apoptosis, cancer stem cells, colonospheres, spheroids, colitis associated cancer, hippo signaling, leucine-rich repeat-containing G-protein coupled receptor 5, doublecortin-like kinase 1

## Abstract

Honokiol (HNK) is a biphenolic compound that has been used in traditional medicine for treating various ailments, including cancers. In this study, we determined the effect of HNK on colon cancer cells in culture and in a colitis-associated cancer model. HNK treatment inhibited proliferation and colony formation while inducing apoptosis. In addition, HNK suppressed colonosphere formation. Molecular docking suggests that HNK interacts with reserve stem cell marker protein DCLK1, with a binding energy of −7.0 Kcal/mol. In vitro kinase assays demonstrated that HNK suppressed the DCLK1 kinase activity. HNK also suppressed the expression of additional cancer stem cell marker proteins LGR5 and CD44. The Hippo signaling pathway is active in intestinal stem cells. In the canonical pathway, YAP1 is phosphorylated at Ser127 by upstream Mst1/2 and Lats1/2. This results in the sequestration of YAP1 in the cytoplasm, thereby not allowing YAP1 to translocate to the nucleus and interact with TEAD1-4 transcription factors to induce gene expression. However, HNK suppressed Ser127 phosphorylation in YAP1, but the protein remains sequestered in the cytoplasm. We further determined that this occurs by YAP1 interacting with PUMA. To determine if this also occurs in vivo, we performed studies in an AOM/DSS induced colitis-associated cancer model. HNK administered by oral gavage at a dose of 5mg/kg bw for 24 weeks demonstrated a significant reduction in the expression of YAP1 and TEAD1 and in the stem marker proteins. Together, these data suggest that HNK prevents colon tumorigenesis in part by inducing PUMA-YAP1 interaction and cytoplasmic sequestration, thereby suppressing the oncogenic YAP1 activity.

## 1. Introduction

Colon cancer is a second leading cause of adult cancer related death in the United States and is associated with a high mortality rate [1]. Patients with inflammatory bowel disease (IBD) are at increased risk of developing colorectal cancer. In particular, an association between inflammatory bowel disease and the development of colitis-associated cancer (CAC) has been reported [2]. Worldwide, the disease will also affect one in three men and one in four women during their lifetime [3]. The American Cancer Society estimates that in 2021, 101,270 new cases (52,590 men and 51,680 women) would be diagnosed with colon cancer, and of these 52,980 would result in death (28,520 men and 24,460 women) [1]. While current chemotherapy, including a combination of 5-flurouracil, oxaliplatin, and irinotecan coupled with surgical and radiation therapy, shows an initial response, it is often inadequate and results in severe side effects. Hence, there is an urgent need for new treatment regimens. Although novel targeted agents and combinations have been identified, these have not been very effective or curative in patients with advanced cancer.

Honokiol (HNK) is a biphenolic compound present in the leaves, bark, and root of the Magnolia officianalis plant. The plant has been used in traditional Chinese and Japanese medicine for the treatment of various ailments, including ulcers, allergies, and bacterial infections. Several studies have demonstrated that it has a multifunctional anti-tumor activity and a low toxicity [4,5,6]. We previously demonstrated that HNK in combination with radiation targets notch signaling to inhibit colon cancer and melanoma stem cells [7]. Recent studies have also shown that HNK eliminated oral cancer stem-like cells accompanied by the suppression of Wnt/*β* catenin signaling [8]. In addition, HNK potentiated the temozolomide sensitivity in glioblastoma multiforme cells in a similar manner [9]. In addition, studies have shown that HNK inhibits epithelial–mesenchymal transition and suppresses stemness [10,11].

Cancer stem cells (CSCs) are highly resistant to standard therapy. Hence, developing therapeutic agents that target these cells, while not affecting normal stem cells, is especially important [12]. Multiple markers have been identified that are present in normal intestinal and cancer stem cells, including, but not limited to CD44, CD24, CD166, CD133, telomerase (TERT), doublecortin calmodulin-like kinase 1 (DCLK1), and LGR5. Moreover, serial isolation of cells using cell surface markers CD44 and CD24, and finally with the Aldefluor assay for ALDH, have been used for enriching CSCs [13]. LGR5 is a normal stem cell marker that is present in highly proliferating cells [14]. We have demonstrated that DCLK1 marks the +4 tuft cells also serves as a reserve stem cell marker when LGR5+ cells are removed as a result of injury [15,16]. Moreover, studies have confirmed that DCLK1 distinguishes between tumor and normal stem cells in the intestine, and the specific ablation of DCLK1+ CSCs results in a marked regression of polyps without apparent damage to the normal intestine. It could therefore be a potential therapeutic target for colorectal cancer [17].

The Hippo cascade, initially identified in Drosophila, is a novel signaling pathway pivotal for normal cellular development and organ size control [18,19]. There are two sets of core kinases, Mst1/2 and Lats1/2, whose activity is modulated by Sav and Mob. When the pathway is active, the phosphorylation of the Yes-associated protein 1 (YAP1) or TAZ by Lats1/2 results in inhibiting nuclear translocation and subsequent cytoplasmic degradation. On the other hand, when the pathway is inactivated, YAP/TAP is allowed to enter the nucleus, where it interacts with TEAD family members to activate transcription. In the nucleus, the disruption of the YAP-TEAD complex can be achieved by compounds such as the porphyrin molecule verteporfin [20]. Here, verteporfin binds YAP1 and changes its conformation to inhibit its interaction with TEAD. Dysregulation of the pathway is implicated in many types of cancers; for instance, YAP1/TAZ and TEAD are upregulated in a variety of human tumors by mechanisms that include gene amplification and silencing of upstream components of the pathway. FAT4 is mutated in 5% of patients with gastric cancer [21], while the SAV1 gene is mutated in colon and renal cancers [22]. Furthermore, a reduced MST1/2 and LATS1 expression was found in gastric cancer, and tumors with lymph node metastasis showed minimal levels of LATS1 [23,24]. Moreover, YAP1 is overexpressed at a high frequency in many common human cancers, and can directly drive cancer development in mouse models [25,26].

In the canonical pathway, YAP1 phosphorylation at Ser127 results in an interaction with the 14-3-3 protein, leading to cytoplasmic sequestration and subsequent ubiquitination-proteasomal degradation of the protein. However, in the current manuscript, we demonstrate that HNK suppresses YAP1 phosphorylation at Ser127. Nevertheless, YAP1 is sequestered in the cytoplasm through interaction with PUMA. This is similar to previous studies showing PUMAs interaction with Bcl2 and inhibiting the Bcl2 function [27,28]. The result of this interaction is the suppression of stemness and the induction of apoptosis.

## 2. Materials and Methods

### 2.1. Cell Lines and Reagents

HCT116, SW480, DLD1, LS174T, RKO, and HT29 human cancer cells (American Type Culture Collection, Manassas, VA, USA) were grown in DMEM containing 10% heat-inactivated fetal bovine serum (Sigma Chemical Co, St. Louis, MO, USA) and 1% antibiotic-antimycotic solution (Mediatech Inc, Herndon, VA, USA) at 37 °C in a humidified atmosphere containing 5% CO_2_. HNK (Catalog No.H5654) was purchased from LKT Laboratories, St Paul, MN, USA.

### 2.2. Proliferation and Apoptosis Assays

The cells were seeded onto 96 well plates and were grown overnight. After treating with increasing doses of HNK (0–50 µM) in 10% FBS containing DMEM, the cell proliferation was analyzed using a hexosaminidase assay [29,30]. For apoptosis, the caspase 3/7 activity was measured using the Apo-one Homogeneous Caspase-3/7 Assay kit (Promega, Madison, WI, USA).

### 2.3. Clonogenicity Assay

Briefly, six-well dishes were seeded with 500 viable cells per well, treated with HNK (25 µM) in 10% FBS containing DMEM for 48 h, the HNK-containing medium was removed, and the cells were incubated for an additional 10 days. The colonies obtained were fixed in formalin and stained with crystal violet, and were subsequently counted. The number of colonies following treatment was compared with the untreated cells.

### 2.4. Cell Cycle Analyses

The cells were treated with HNK for 12, 24, and 48 h, and were subsequently suspended in PBS following trypsinization. The cells were fixed with 70% ethanol overnight and were subsequently permeabilized at room temperature in PBS containing 1 mg/mL propidium iodide (Sigma-Aldrich, St. Louis, MO, USA), 0.1% Triton X-100 (Sigma-Aldrich, St. Louis, MO, USA), and 2 µg DNase-free RNase (Sigma-Aldrich, St. Louis, MO, USA). Flow cytometry was performed with a FACSCalibur analyzer (Becton Dickinson, Mountain, View, CA, USA), capturing 50,000 events for each sample, and was subsequently analyzed with ModFit LT TM software 2.0 (Verity Software House, Topsham, ME, USA).

### 2.5. Real-Time Reverse-Transcription Polymerase Chain Reaction Analysis

The total RNA isolated from the cells using a TRIZOL reagent was reverse transcribed with Superscript II reverse transcriptase in the presence of random hexanucleotide primers (Invitrogen, Carlsbad, CA, USA). Real time PCR was performed using Jumpstart Tag DNA polymerase (Sigma Aldrich, St. Louis, MO, USA) and SYBR Green nucleic acid stain (Molecular Probes, Eugene, OR, USA). The crossing threshold values for individual genes were normalized to *β*-Actin. Changes in the mRNA expression were expressed as the fold change relative to the control. The primers used in this study were as follows: *β*-Actin: 5′-GCTGATCCACATCTGCTGG-3′ and 5′-ATCATTGCTCCTCCTCAGCG-3′; DCLK1: 5′- CAGCAACCAGGAATG-TATTGGA-3′, and 5′ CTCAACTCGGAATCGGGAAGACT-3′; LGR5: 5′-TGCTGGCTGGTGTGGATGCG-3′ and 5′-GCCAGCAGGGCACAGAGCAA-3′; CD44: 5′-CAATAGCACCTTGCCCACAAT-3′ and 5′-AATCACCACGTGCCCTTCTATGG-3′; p53: 5′-CCTCAGCATCTTATCCGAGTGG-3′ and 5′-TGGATGGTGGTACAGTCAGAGC-3′; and PUMA: 5′-ACGACCTCAACGCACAGTACGA-3′ and 5′-CCTAATTGGGCTCCATCTCGGG-3′.

### 2.6. Flow Cytometric Analyses for DCLK1

After 24 h following HNK (25 µM) treatment, the cells were harvested and suspended in PBS containing 0.5% BSA for 10 min at room temperature, followed by the addition of 10 μL phycoerythrin-conjugated DCLK1 antibody (Abcam Inc, Cambridge, MA, USA) at room temperature for 1h dark in a rotator. The samples were analyzed using a FACS Calibur analyzer (Becton Dickinson, Mountain, View, CA, USA), capturing 10,000 events for each sample. The results were analyzed with ModFit LT TM software (Verity Software House, Topsham, ME, USA).

### 2.7. Colonosphere Assay

The cells were cultured in DMEM supplemented with 20 ng/mL bFGF 10 mL per 500 mL of 50X B27 supplement, EGF 20 ng/mL (all from Invitrogen, Carlsbad, CA, USA) at low densities (5000 cells/mL) in six-well low adhesion plates. The cells were treated with HNK (25 µM). After 5 days, the number and size of colonospheres were determined.

### 2.8. DCLK1 Kinase Assay

Purified DCLK1 kinase domain (1.5 μg, Signalchem, Richmond, BC, USA) was incubated in a reaction buffer (Invitrogen, Carlsbad, CA, USA) with DMSO, 10, 20, 60, or 120 µM HNK for 30 min at 37 °C. Consequently, 100 μL of luciferin−luciferase mixture (ATP determination kit, Invitrogen) was added into each well, and the luminance was monitored at 560 nm using a SynergyTM NEO microplate reader.

### 2.9. Molecular Docking

The docking study was done using AutoDock Vina Program [31] to predict the interaction of HNK with DCLK1. The kinase domain of DCLK1 PDB ID (5JZN.pdb) protein was downloaded from the Protein Data Bank (RCSB PDB) online database on 22 January 2021 (www.rcsb.org/pdb). The ligand molecules in the active site were removed and the 3-D grid box was created with grid center coordinates and 60 × 60 × 60-point size covering all of the active site residues. The studies used default parameters of the Autodock Tools. Before docking, the protein was prepared by adding hydrogens, total Kollman, and Gasteiger charges. To obtain the best confirmations, Lamarckian GA was utilized. About 10 conformations for HNK docked in the DCLK1 kinase domain were generated, of which the most stable conformation was selected based on the predicted lowest binding energy and the number of hydrogen bonds. The DCLK1-HNK docking conformation was visualized on 22 January 2021 with PyMOL 2.5 (https://pymol.org/2/) software [32].

### 2.10. Immunoprecipitation

Briefly, HCT116 and SW480 cytoplasmic extracts were incubated with the indicated antibody (anti-YAP1 antibody) or an IgG negative control for 3 h at 4 °C in a rotator. Subsequently, agarose beads were added, and incubation continued overnight. The beads were pelleted, washed three times, and then dissolved in a gel loading buffer and analyzed with SDS-PAGE.

### 2.11. Western Blot Analysis

The cell lysates were subjected to polyacrylamide gel electrophoresis and blotted onto Immobilon polyvinylidene difluoride membranes (Millipore, Bedford, MA, USA). The antibodies were purchased from Cell Signaling Technology (Beverly, MA, USA), Abcam Inc. (Cambridge, MA, USA), and Santa Cruz Biotechnology Inc. (Santa Cruz, CA, USA), and the specific proteins were detected by the enhanced chemiluminescence system (GE Health Care, Piscataway, NJ, USA). Western blot images were quantified using ImageJ software (Fiji-win64, 26 May 2021).

### 2.12. Mouse Model of Colitis-Associated Cancer

The CAC model was induced as described previously [33] using azoxymethane (AOM; Sigma-Aldrich, St. Louis, MO, USA) and dextran sulfate sodium (DSS; Affymetrix, Santa Clara, CA, USA). Five-week-old male ICR mice (CD-1) purchased from Charles River Laboratory were utilized for the in vivo experiments. They were maintained with water and standard mouse chow ad libidum, and the protocols used were approved by the University’s Animal Studies Committee. The ICR mice were injected intraperitoneally with 10 mg/kg body weight of AOM followed by three cycles of 2.5% DSS. Subsequently, HNK (5 mg/kg body weight mixed in 0.5% methyl propyl hydroxyl cellulose in water with 0.9% Tween 80) was administered orally three days a week for 24 weeks. The animals were euthanized at the end of the study, and the tumors were excised and used for immunohistochemistry.

### 2.13. Immunohistochemistry

Paraffin-embedded tissues were cut into 4 µm sections, deparaffinized, and blocked with Avidin/Biotin for 20 min. The slides were incubated with primary antibodies overnight at 4 °C. Next the slides were treated with broad-spectrum secondary antibody (Invitrogen, Carlsbad, CA, USA) and HRP-conjugate for 1 h and were then developed with DAB (Invitrogen, Carlsbad, CA, USA). Finally, the slides were counterstained with hematoxylin. The slides were examined in a Nikon Eclipse Ti microscope under a 40× objective.

### 2.14. Statistical Analysis

All of the values are expressed as the mean ±SEM. Data were analyzed using an unpaired 2-tailed *t*-test. A *p* value of less than 0.05 was considered statistically significant.

## 3. Results

### 3.1. HNK Inhibits Colon Cancer Cell Proliferation and Colony Formation

**Figure 1:**
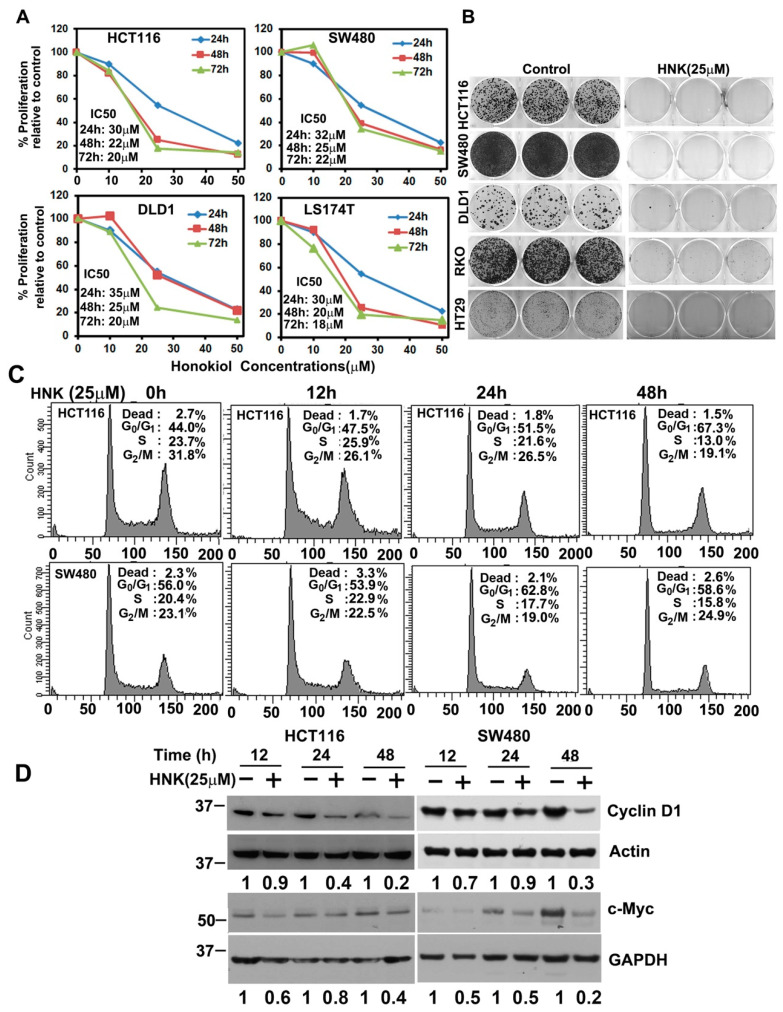
Honokiol (HNK) inhibits colon cancer cell growth. (**A**) HCT116, SW480, DLD1, and LS174T cells were incubated with increasing doses of HNK (0–50 μM), and cell proliferation was determined for up to 72 h. HNK treatment resulted in a significant dose- and time-dependent decrease in cell proliferation in all four cell lines when compared with the controls. (**B**) Cells were incubated with 25 µM HNK for 48 h and subsequently allowed to grow and form colonies in regular media. HNK treatment inhibited colony formation. (**C**) Cell cycle analysis. HCT116 and SW480 cells were treated with 25 µM HNK for up to 48 h and then examined by flow cytometry following propidium iodide staining for DNA content. HNK treatment induced G0/G1 arrest. (**D**) Lysates from cells incubated with 25 µM HNK for up to 48 h were analyzed by Western blotting for cyclin D1 and c-Myc expression. HNK inhibits the expression of both cyclin D1 and c-Myc in HCT116 and SW480 cells.

First, we determined the effect of HNK on the cell proliferation using the hexosaminidase assay [29]. HNK treatment suppressed the proliferation of all four cell lines in a dose- and time-dependent manner. While the anti-proliferation effect was observed within 24 h, it continued to further suppress proliferation for the next 72 h (Figure 1A). To further determine whether HNK treatment had a long-term effect on the cell lines, we treated then with HNK for 48 h and then allowed them to grow and develop in a normal medium. Because of the HNK treatment, the colony numbers reduced in all five cell lines, namely, HCT116, SW480, DLD1, RKO, and HT29 cells, when compared with the untreated control (Figure 1B). Given that HNK inhibits proliferation and colony formation, we next determined whether HNK affects cell cycle progression. Treatment with HNK significantly induced G1 arrest in both HCT116 and SW480 cells at 24 and 48 h (Figure 1C). Studies have suggested that when the cell cycle is perturbed at the G1/S transition, it leads to cancer development. A major cellular factor that governs the transition is cyclin D1, which is in complex with CDK4 or CDK6. In addition, cyclin D1 is a cofactor for several transcription factors [30]. Therefore, not surprisingly, cyclin D1 overexpression potentially plays a role in cancer development and progression [31]. Cyclin D1 has been shown to be regulated by c-myc, a cellular protooncogene, which in turn affected cell cycle progression [34]. In both HCT116 and SW480 cells, HNK treatment resulted in reduced cyclin D1 and c-Myc expression (Figure 1D).

### 3.2. HNK Prevents Colitis-Associated Cancer Growth

**Figure 2:**
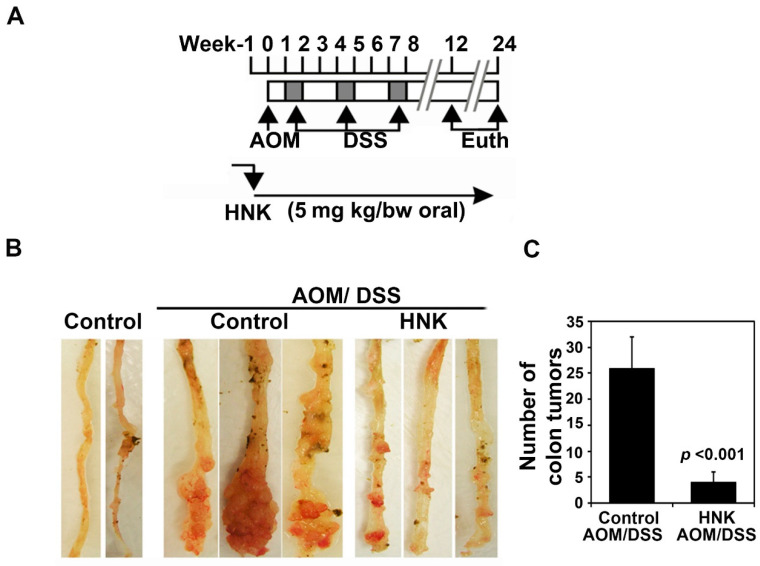
HNK prevents colitis-associated cancer. (**A**) Experimental plan. The ICR mice were injected intraperitoneally with 10 mg/kg body weight azoxymethane (AOM), followed by three cycles of 2.5% dextran sulfate sodium (DSS). HNK (5mg/kg body weight mixed in 0.5% methyl propyl hydroxyl cellulose in water with 0.9% Tween 80) was administered orally three days a week for 24 weeks. The animals were sacrificed at the end of the study and the colons were excised and subject to further analyses. (**B**) HNK prevents colitis-associated cancer (CAC) tumors when compared with the control AOM/DSS group. (**C**) The colonic tumors were counted. HNK treatment significantly reduced the colonic tumor numbers and size when compared with the AOM/DSS control (*p* < 0.001).

To evaluate the preventive role of HNK on CAC growth in vivo, we next determined its preventive effects on AOM/DSS CAC growth. The ICR mice were injected intraperitoneally with 10 mg/kg bw of AOM, followed by three cycles of 2.5% DSS. Subsequently, HNK (5 mg/kg body weight mixed in 0.5% methyl propyl hydroxyl cellulose in water with 0.9% Tween 80) was administered orally three days a week for 24 weeks (Figure 2A,B). HNK prevented CAC tumors (Figure 2B). While there were high numbers of colonic tumors in the AOM/DSS group alone that were also large in size, those treated with HNK had fewer tumors and the size of the tumors was smaller (Figure 2C). There was no apparent change in the liver, spleen, or body weight in the animals (data not shown).

### 3.3. HNK Treatment Induces Apoptosis

**Figure 3:**
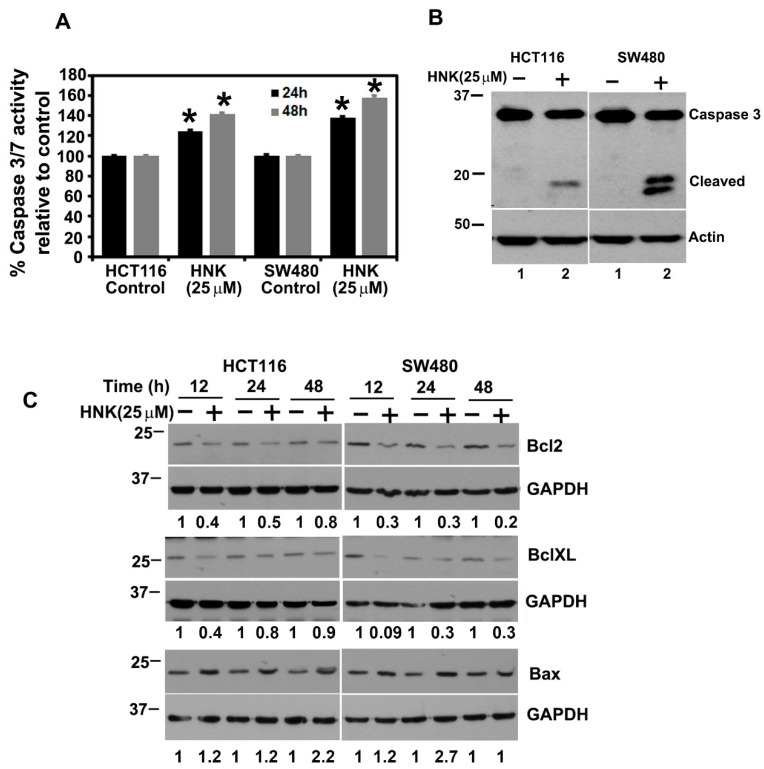
HNK induces apoptosis. (**A**) HCT116 and SW480 cells were treated with 25 µM HNK for 48 h and were tested for caspase-3 and -7 activity. HNK treatment induces the activity in both cells, when compared with the control (* *p* < 0.01). (**B**) HNK induces caspase 3, an apoptosis mediator. The cells were treated with 25 μM HNK and after 48 h, and lysates were analyzed by Western blotting for the caspase 3 protein. The HNK treatment resulted in increased levels of cleaved caspase 3 when compared with the control. (**C**) Lysates from cells incubated with 25 µM HNK for up to 48 h were analyzed by Western blotting for the Bcl2, BclXL, and Bax expression. HNK inhibits Bcl2 and BclXL, while increasing the Bax expression in both HCT116 and SW480 cells.

Given that HNK inhibits colony formation and induces G1 arrest, we next determined whether HNK induced apoptosis. Caspases are a family of proteins known for mediating apoptotic cell death. Caspase-3 and -7 are key effector molecules known to induce apoptosis in a variety of cancer cells by amplifying the signal from the initiator caspases, such as caspase 8 or caspase 10 [35,36]. Increased activation of effector caspases 3 and 7 were observed in 24 and 48 h in both the HCT116 and SW480 cells treated with HNK, suggesting the induction of apoptosis (Figure 3A). Western blot analyses also demonstrated a significant increase in cleaved caspase-3 in HNK treated tumor cells at 48 h (Figure 3B). We next confirmed that HNK induces apoptosis by performing Western blot analyses for the anti-apoptotic proteins Bcl2 and BclXL, and the pro-apoptotic protein Bax. HNK treatment reduced Bcl2 and BclXL levels, whereas there was a significant increase in Bax protein levels in both the HCT116 and SW480 cells (Figure 3C). These data suggest that HNK induces apoptosis.

### 3.4. HNK Inhibits Cancer Stem Cell Marker DCLK1 Expression in Both In Vitro and Colonic Tumors

**Figure 4:**
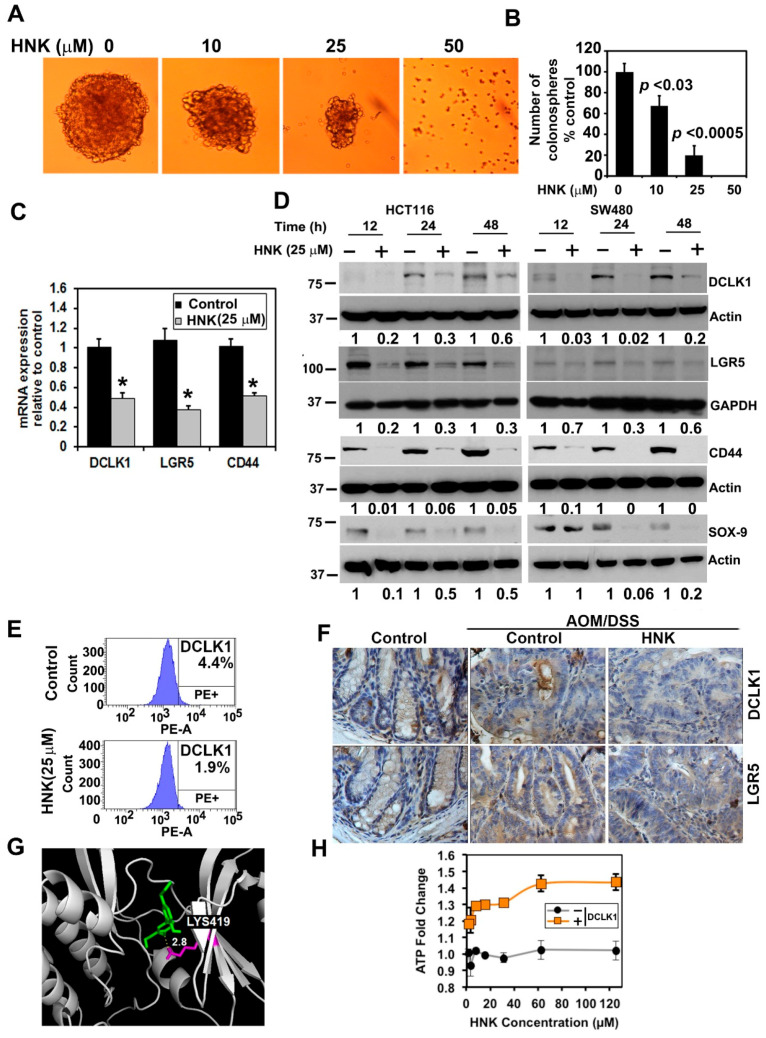
HNK affects stem cell marker expression. (**A**) HCT116 cells were grown in spheroid growth media in low adherent plates and treated with 25 µM HNK. After 5 days, the colonospheres were photographed and counted. (**B**) HNK treatment significantly inhibited colonosphere formation (*p* < 0.03 or 0.0005). (**C**) Total RNA from HCT116 cells treated with HNK for 24 h was subjected to real-time PCR analyses. HNK treatment showed a significant reduction in the expression of DCLK1, LGR5, and CD44 mRNA (* *p* <0.05). (**D**) Western blot analyses of the lysates from the HNK treatment showed a significant reduction in cancer stem cell marker protein levels in both HCT116 and SW480 cells. (**E**) Sorting of anti-DCLK1 and antibodies tagged untreated SW480 cells by flow cytometry. After 24 h, HNK treatment resulted in a significant reduction in the number of cells expressing DCLK1 on the surface. (**F**) Immunohistochemistry shows that treatment with HNK significantly reduced the expression of cancer stem cell marker proteins DCLK1 and LGR5. (**G**) HNK interacts with the DCLK1 kinase domain. Homology modeling shows that HNK can interact with the kinase domain with a binding energy of −7.0 Kcal/mol. (**H**) In vitro kinase assay shows that HNK treatment inhibits DCLK1 kinase activity.

As HNK treatment suppresses the growth of colon cancer cells in a two-dimensional culture, we next determined the effects of HNK on the colonosphere formation assay. Here, cells were grown in ultra-low binding plates so that they could aggregate and grow to form spheroids. In this assay, HNK significantly inhibited HCT116 colonosphere formation (Figure 4A,B). This suggests that HNK treatment suppresses the growth of colon cancer cells in both two-dimensional and three-dimensional cultures. To confirm the HNK effect on CSCs, we determined the effect of HNK on the expression of stem cell marker proteins. Following HNK treatment, there were significantly low levels of transcripts encoding DCLK1, LGR5, and CD44 mRNA (Figure 4C). Western blot analyses confirmed these findings showing a reduction in their protein levels, as well as that of SOX-9 (Figure 4D). Flow cytometric analyses also showed a decrease in DCLK1+ in SW480 cells following HNK treatment (Figure 4E). Next, we investigated whether HNK affects CSCs by determining the expression of DCLK1 and LGR5 in tumor tissues. Immunohistochemistry analyses demonstrated that in tumors obtained from animals administered HNK, there was a reduction in the expression of DCLK1 and LGR5 when compared with the controls (Figure 4F). Given that there was reduction in stem cells, we next determined whether HNK directly affects stem cell marker proteins. DCLK1 is a microtubule-associated kinase protein. The kinase domain is located in the C-terminus of the protein and has homology to calmodulin-dependent kinases of the CAMKI family, although it lacks the calmodulin binding site [37]. As the kinase domain of DCLK1 is crystallized, we used the structure to perform in silico modeling. HNK was identified to interact with LYS419 by forming a hydrogen bond within the kinase domain with a binding energy calculated to be −7.0 kcal/mol (Figure 4G). Next, we determined if HNK affects the DCLK1 kinase activity. For this, we performed an in vitro kinase assay using recombinant DCLK1. Again, HNK significantly reduced the DCLK1 kinase activity (Figure 4G). These data suggest that HNK targets DCLK1, thereby suppressing colon CSCs.

### 3.5. HNK Inhibits Hippo Signaling through Downregulation YAP1 in Both In Vitro and Colonic Tumors

**Figure 5:**
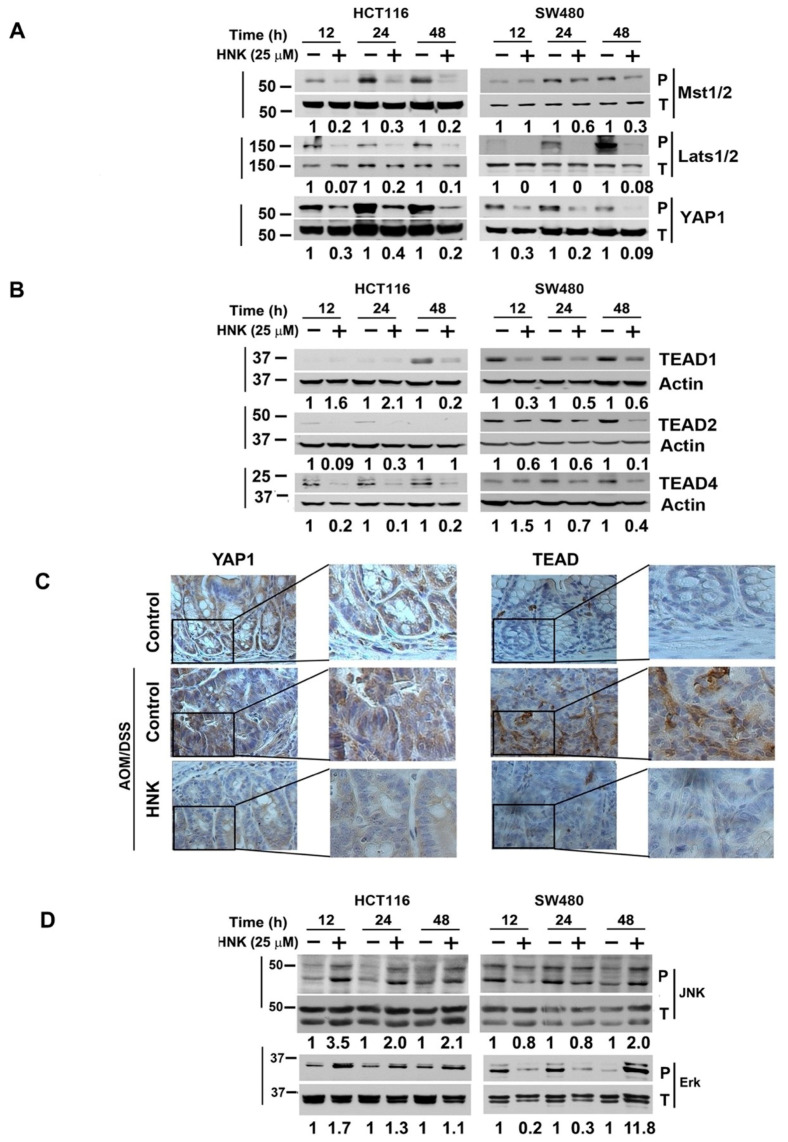
HNK inhibits Hippo signaling. (**A**) Western blot analyses of lysates from HNK treated cells show a significant inhibition of the phosphorylation of Mst1/2, Lats1/2, and YAP1 using phospho-specific antibodies. (**B**) HNK treatment resulted in a reduced expression of TEAD1, 2, and 4 in both HCT116 and SW480 cells. (**C**) Immunohistochemistry shows that the HNK treated animals have lower levels of YAP1 and TEAD1 in the CAC tissues. (**D**) Lysates from HNK treated cells caused a significant increase in the phosphorylation of JNK and ERK1/2.

Dysregulation of the Hippo signaling pathway is implicated in many types of cancers; for instance, YAP1/TAZ and TEAD are upregulated in a variety of human tumors by mechanisms that include gene amplification and silencing of upstream components of the pathway. YAP1 can also directly drive cancer development in mouse models [25,26]. In addition, YAP1 has been shown to control the stem cell fate [38,39]. We determined the effect of HNK on Hippo signaling proteins in HCT116 and SW480 cells. HNK treatment reduced the phosphorylation of Mst1/2, Lats1/2, and YAP1 using phospho-specific antibodies (Figure 5A). Moreover, HNK treatment reduced the expression of TEAD1, TEAD2, and TEAD4 in both cell lines (Figure 5A,B). We also examined the effects of HNK on YAP1 and TEAD1 expression in the tumor tissues from the AOM/DSS treated mice. Treatment with the HNK resulted in significantly reduced levels of YAP1 and TEAD1 when compared with the control untreated tumors (Figure 5C). These data imply that HNK is a potential preventive agent for treating colon cancers, in part by affecting expression of Hippo signaling oncogenic protein YAP1 and TEAD1 proteins. Previous studies have demonstrated that JNK phosphorylates YAP1, and this leads to apoptosis [40]. In addition, HNK can activate ROS/ERK1/2 signaling to induce apoptosis [41]. Accordingly, we determined the effect of HNK on JNK and ERK1/2 phosphorylation. However, treatment with HNK induced the phosphorylation of both proteins in the two cell lines, especially after 48 h of treatment (Figure 5D).

### 3.6. HNK Downregulates YAP1 through PUMA Mediated Apoptosis

**Figure 6:**
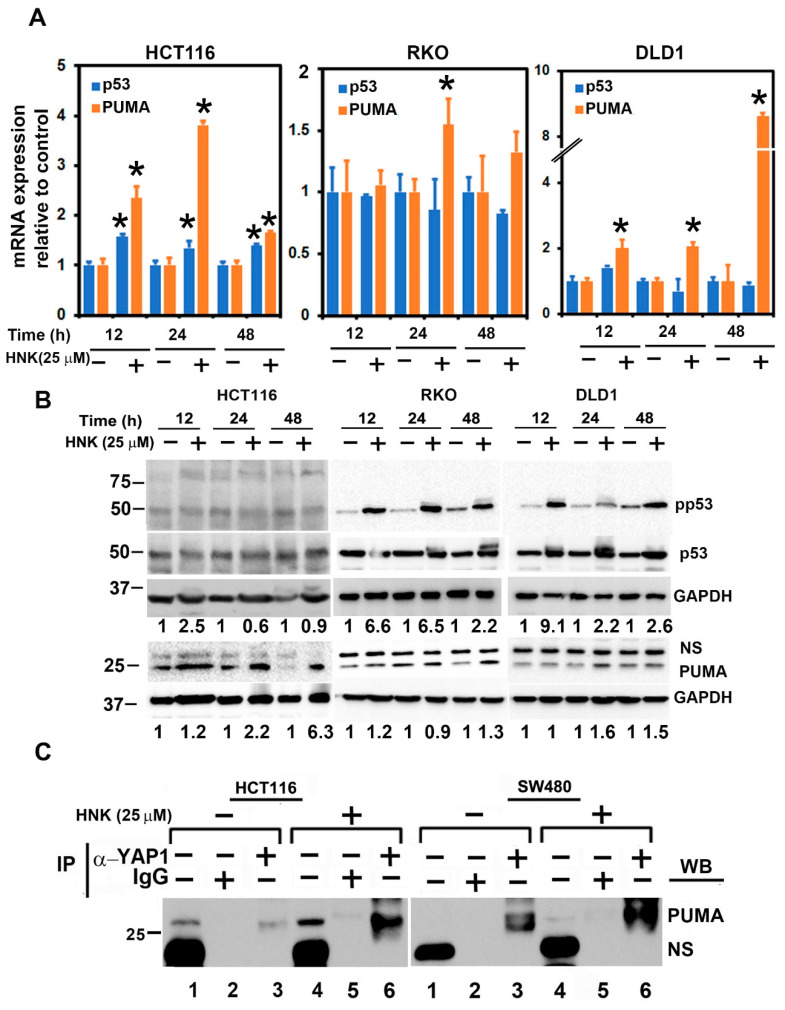
HNK downregulates and induces the PUMA:YAP1 interaction in the cytoplasm. (**A**) Total RNA from cells treated with HNK for up to 48 h was subjected to real-time PCR analyses. HNK treatment showed a significant increase in the expression of p53 and PUMA mRNA (* *p* < 0.05). (**B**) Lysates from HNK treated cells show an increase in p53 phosphorylation and PUMA expression. (**C**) Cytoplasmic extracts from HNK treated cells were immunoprecipitated with YAP1, and subsequently the immunoprecipitate was analyzed by Western blotting for PUMA. HNK treated cytoplasmic extracts show increased binding of PUMA in HNK treated cells when compared with untreated cells.

Previous studies have demonstrated that MEKK1-JNK signaling can increase the stability, transcriptional activities, and apoptotic capacity of the p53 protein [42]. PUMA (p53-upregulated modulator of apoptosis) is a member of the Bcl-2 family of proteins that was identified as a p53 target and could be a direct mediator of p53-associated apoptosis [43]. Given the effects of JNK, we determined whether the expression of p53 or PUMA is affected. The total RNA from HCT116 cells treated with HNK were subjected to quantitative PCR. There was significant upregulation for both genes for up to 24 h (Figure 6A). We confirmed the upregulation at the protein level in RKO and DLD1 using Western blot analyses. In all of these cells, HNK treatment increased the induced PUMA expression (Figure 6B). In addition, there was not only an induction on a p53 levels, but also increased phosphorylation of the protein.

In the canonical pathway, YAP1 phosphorylation at Ser127 resulted in an interaction with 14-3-3 protein, leading to cytoplasmic sequestration and subsequent ubiquitination-proteasomal degradation [44]. However, following HNK treatment, there was no phosphorylation observed at Ser127. Studies have shown that PUMA interacts with Bcl2 and inhibits Bcl2 function [27,28]. Moreover, studies have shown that PUMA is localized in the cytoplasm, and that cytoplasmic accumulation of the protein may be important for the apoptotic process [45]. Accordingly, we determined if PUMA interacts with YAP1. For this, we generated cytoplasmic extracts from control and HNK treated cells, and performed immunoprecipitation for YAP1 followed by Western blot for PUMA. We observed that the PUMA:YAP1 interaction in the control, untreated cells was significantly increased following HNK treatment (Figure 6C). These data suggest, for the first time, that in the absence of Ser127 phosphorylation of YAP1, the protein can be sequestered in the cytoplasm by PUMA, a novel mechanism of inhibiting YAP1 function.

## 4. Discussion

HNK, a natural compound, has been shown to have anticancer activities in various cells, including skin, colon, lung, breast, and pancreatic cancers. HNK has exhibits a desirable spectrum of bioavailability, and significant systemic levels of HNK can be observed in the blood in preclinical models. In addition, HNK can cross the blood–brain barrier [5]. Currently, it is not known whether HNK has a single major target or several targets. However, it has several activities that make it desirable as both a therapeutic and chemopreventive agent. These studies suggest that HNK could be used as an effective agent for chemotherapeutic drugs.

HNK can affect many of the characteristic cancer-promoting events, including cancer stem cells, through suppressing the Notch signaling pathway [7,46]. Cancer stem cells have been shown to be regulated by multiple signaling pathways, including the Wnt, Notch, Hedgehog, and Hippo pathways [47]. These pathways in CSCs can also have the capacity to drive tumor resistance and recurrence to chemotherapeutic agents [48]. Natural compounds such as curcumin, sulforaphane, quercetin, and piperine can all target CSCs [49,50]. Similarly, our results suggest that HNK is a potent inhibitor of CSCs. A commonly used method to demonstrate stemness is by growing spheroids in ultra-low binding tissue culture dishes. HNK treatment significantly inhibited colonosphere formation. We have also determined that HNK interacts with the DCLK1 kinase domain through homology modeling. Given that DCLK1 expressing cells play a significant role in tumor generation, the targeting of this protein by HNK would be a significant mechanism for suppressing tumorigenesis. Additional markers such as LGR5 and SOX9 have also been identified as stem cell marker proteins [51,52]. Indeed, HNK was found to suppress the expression of these proteins, as well further demonstrating that the compound targets CSCs. This was also confirmed in vivo, where HNK treatment significantly reduced the expression of DCLK1 and LGR5 in the colonic tumors.

**Figure 7:**
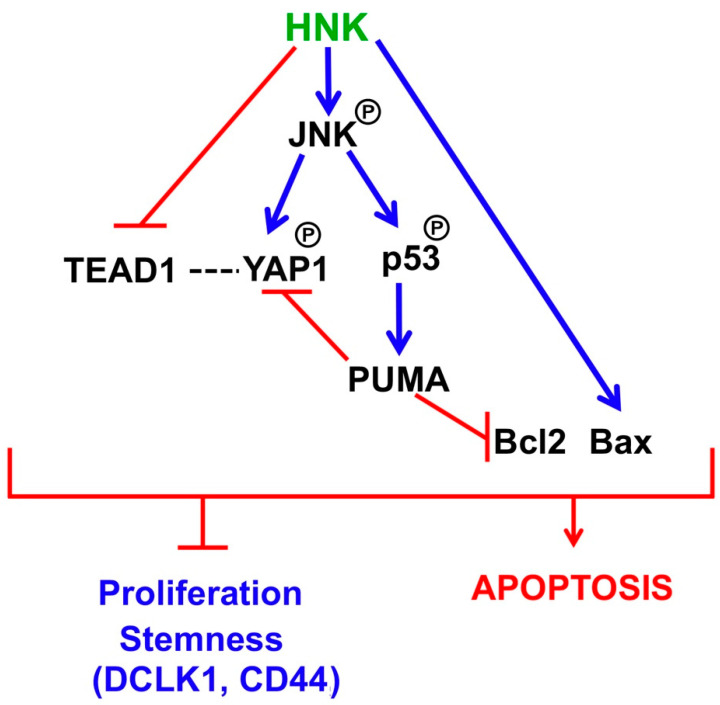
Schematic representation showing that HNK inhibits colonic tumorigenesis and affects YAP1 function in stem cells through PUMA.

More recently, the Hippo signaling pathway has been gaining recognition as an important player in tumorigenesis because of the disruption to several components, including Mst1/2, Sav1, and Lats1/2, and YAP1 in the pathway [53]. YAP1, which is the effector in the pathway, has oncogenic properties in several tumors [54,55]. In the canonical pathway, YAP1 is phosphorylated at Ser127 by upstream serial activation of Mst1/2 and Lats1/2, and is then sequestered in the cytoplasm, where it interacts with 14-3-3 proteins and gets degraded. YAP1 is a nuclear protein, where it interacts with TEAD proteins to activate the target gene expression. In transgenic mice, tissue-specific expression of YAP1 results in tissue overgrowth and tumor formation [53]. The Hippo signaling pathway is also active in intestinal stem cells [56]. We have observed that HNK treatment reduces YAP1 phosphorylation and suppresses the expression of TEAD1, 2, and 4. However, this does not occur through the canonical pathway, because HNK inhibits Ser127 phosphorylation, a first step required for YAP1 sequestration and degradation in the cytoplasm. However, we believe an additional pathway for YAP1 sequestration exists that involves JNK-PUMA. Activated JNK has been previously shown to increase YAP1 phosphorylation at Ser138, Ser317, and Thr362 [40]. Furthermore, JNK has been shown to play a role in transcriptionally inducing PUMA gene expression [57]. We observed JNK activation in response to HNK. In addition, we observed the activation of p53 and the induction of the PUMA expression. PUMA, in turn, bound to YAP1 in the cytoplasm. This is very similar to the previous reported activity of PUMA, wherein its interaction with Bcl2 suppresses Bcl2 function [27,28]. Therefore, we believe that HNK functions to suppress the expression of TEAD1, while inducing that of PUMA to suppress YAP1 and Bcl2. In addition, HNK induces the Bax expression (Figure 7). The cumulative effect of all of these activities is the induction of apoptosis and the suppression of stemness and tumorigenesis. Another question that has been raised by these studies is whether there is any connection between Hippo signaling and stem cell marker expression. It is intriguing to consider that the pathway may play a role in the expression of stem cell markers such DCLK1 and LGR5. Given that DCLK1 and LGR5 are markers of a reserve, stress-induced and active, cycling cancer stem cells, respectively, our future studies will focus on looking at the role of the pathway in differentially regulating these two stem cell populations.

## 5. Conclusions

In conclusion, the current studies provide evidence that the prevention/treatment of colon cancer with HNK results in growth inhibition in in vitro and in vivo CAC cancer models. Furthermore, HNK treatment was more potent in colon CSCs, based on the marker expression and inhibition of colonosphere formation. In addition, the HNK significantly suppressed oncogenic YAP1 phosphorylation and TEAD protein expressions. Taken together, these data suggest that HNK is a potent inhibitor of colon CSCs and is a potential agent for the prevention of CAC colon cancers.

## Data Availability

No data being reported.

## Abbreviations

HNK	Honokiol
CSC	Cancer stem cells
LGR5	Leucine-rich repeat-containing G-protein coupled receptor 5
ERK	Extracellular signal-regulated kinase
JNK	Janus kinase
